# Endoparasites in dogs diagnosed at the Veterinary Teaching Hospital (VTH)-University of Bologna, combined with clinicopathological results. A long-term retrospective secondary data study

**DOI:** 10.1371/journal.pone.0293330

**Published:** 2023-10-20

**Authors:** Benedetto Morandi, Maria Chiara Sabetti, Maira Napoleoni, Ilaria Pascucci, Gionata Orlandi, Marco Pietra, John A. VanLeeuwen, Spencer J. Greenwood, Giovanni Poglayen, Roberta Galuppi

**Affiliations:** 1 Istituto Zooprofilattico Sperimentale dell’Umbria e delle Marche “Togo Rosati”, Perugia, Italy; 2 Department of Veterinary Science, University of Parma, Parma, Italy; 3 Department of Veterinary Medical Sciences, *Alma Mater Studiorum*-University of Bologna, Bologna, Italy; 4 Department of Health Management, Atlantic Veterinary College, University of Prince Edward Island, Charlottetown, Canada; 5 Department of Biomedical Sciences, Atlantic Veterinary College, Charlottetown, Canada; National Veterinary Research Institute (NVRI), NIGERIA

## Abstract

Humans and dogs commonly share the same domestic environment. Europe, and Italy specifically, have a substantial and growing dog population. Potentially zoonotic parasites may be harbored even by dogs receiving regular veterinary care. Thus, transmission of zoonotic or potentially zoonotic parasites to owners and their families should not be underestimated. Frequently, endoparasite infections occur as a subclinical infection and clinicopathological alterations have been documented including anemia, hypoalbuminemia, and eosinophilia. The aim of this large retrospective secondary data study was to analyze coprological endoparasite results and putative risk factors obtained from owned dogs, through a 9-year-period (2011–2019). Possible associations between diagnosed endoparasites and sex, age, seasonality, and year of examination were evaluated. Additionally, parasitological diagnoses were combined to complete blood count parameters and biochemical profiles, when available, to check for any possible hematological alteration from parasitism. A total of 1,972 dogs were evaluated for endoparasites using common fecal diagnostic tests over a 9-year period. The overall proportion of endoparasite-positive animals was 10%. The most common endoparasites detected were *Cystoisospora* spp. (3%), *Toxocara canis* (2.8%), *Giardia duodenalis* (1.6%), and *Trichuris vulpis* (1.2%). Of these parasites detected, *Toxocara* poses the greatest zoonotic risk, while *Giardia* species are considered to have a low potential to be zoonotic. There was no significant diagnostic trend across the years through the study period. Dogs were more frequently diagnosed endoparasite-positive when young and during cold seasons compared to the baselines of mature dogs and warm seasons. The clinicopathological profiles indicated that parasitized dogs had mild hematological alterations. The frequency of detected potentially zoonotic endoparasites in this study highlights that the risk should not be underestimated. Parasitic infection was found to be mostly dependent on age and season. Having this information may help clinicians to develop anthelmintic protocols to reduce the risk of transmission.

## Introduction

Dogs live in close contact with owners and often share the same environment and even daily conditions of the human lifestyle. Europe has a substantial and growing dog population; the number of dogs is estimated to be around 90 million [1]. As reported by a reliable on-line portal, the current owned-dog population in Italy is approximately 8.3 million and a human population of around 60.5 million or 14 dogs per 100 people [2]. It is hypothesized that 5.9 million Italian households (27.1%) own dogs which may harbor a variety of potentially zoonotic parasites, even those experiencing routine veterinary care, such as *Toxocara canis*, *Giardia duodenalis*, and *Ancylostoma* spp. [3–5]. Usually, owners consider their dogs as a member of the family [6], having contact and interactions that may produce beneficial physical and social effects on people, but may also directly increase the transmission risk of those zoonotic agents [7]. Working dogs are more commonly diagnosed positive for endoparasites [8] compared to companion animals [3], and the owners may be indirectly infected through contaminated food or water rather than intimate contact [9]. Therefore, veterinarians play a crucial role in zoonotic risk assessment and communication, acting as a main public health professional.

Frequency and prevalence of endoparasites in dogs may change based on season and parasite species, and from region to region within a country [3]. To the best of our knowledge, few published papers have explored seasonal patterns of endoparasitism in dogs in Italy [10], although Morelli et al. had six years of data in their analyses [11].

The most frequently diagnosed dogs’ endoparasites occur as subclinical infection or generally without any specific alterations. Thus, according to ESCCAP (European Scientific Counsel Companion Animal Parasites) guidelines [12], fecal examination should be routinely performed by veterinary clinicians based on the local epidemiological circumstances. Fecal flotation and Baermann technique are probably the most common laboratory procedures performed in veterinary practice. Relatively inexpensive and non-invasive, fecal examination can reveal the presence of parasites in several body systems [13]. Expertise is required to differentiate the parasite species from the many pseudoparasites (such as yeasts, plant remnants, pollens, and debris) that may be mistaken for true parasites. Although it is conceivable that in the near future, fecal flotation slides will be scanned using automated image systems with integrated diagnostic algorithms [14], until then, coprological examination should be carried out carefully and thoroughly by trained veterinary parasitologists or technicians.

The occurrence of clinical signs due to the presence of endoparasites is not common and is influenced by a variety of factors, such as parasite species involved, intensity of infection, host age, nutritional condition, and immunity [15]. Clinicopathological alterations associated with endoparasite infections have been documented and include anemia, hypoalbuminemia, and eosinophilia [16]. However, few studies consider large populations of naturally infected dogs in association with clinicopathological alterations [17].

The study aims to investigate the frequency of endoparasites, and to analyze putative demographic and temporal risk factor data that were available from owned dogs brought to the Veterinary Teaching Hospital (VTH), Department of Veterinary Medical Sciences (DIMEVET), University of Bologna (Unibo), Italy. As an additional objective, parasitological diagnoses were further combined with complete blood count (CBC) parameters and biochemical profiles, when available to check for any possible hematological alteration from parasitism.

Outcomes originated from this study should be an aid for clinician colleagues to better educate their clients about endoparasite occurrence and to use good preventive programs.

## Materials and methods

### Study population and fecal examination

The study population represents the total of the privately owned dogs brought in to the Veterinary Teaching Hospital (VTH), Department of Veterinary Medical Sciences (DIMEVET), University of Bologna (Unibo), who had a fecal sample submitted for parasitological examination, from January 1, 2011 to July 31, 2019. The study population refers to: 1) animals under routine care where the sample was part of a preventive health management plan; or 2) animals exhibiting clinical signs suggestive of parasitism.

For all fecal samples, endoparasite eggs, cysts, and oocysts, were concentrated from fecal specimens by using the Di Felice and Ferretti [18] solution (sodium nitrate and sugar; specific gravity = 1.3) as flotation media in a standardized centrifugal flotation method [19]. Additional methods were employed, including: the Baermann technique for suspected lungworm cases, based on reported clinical signs of coughing and/or dyspnea, to detect the presence of first-stage larvae (L1); and for *Giardia duodenalis* trophozoites by direct smears with Lugol’s iodine staining and cysts by a double sedimentation, based on reported clinical signs of diarrhea [20]. All the parasite elements were identified through existing keys [13, 21, 22].

### Data management and statistical analysis

Data on the dogs were extracted from the management software Fenice® used by the VTH, representing all the dog fecal samples which passed through the Transmissible Diseases and Public Health Service of the DIMEVET for parasitological examination during the study period. Available data in the database included dog sex, age, and geographic origin (based on home address), date of sample submission, diagnostic technique employed (described above), and test results. Additionally, complete blood count (CBC) and serum chemistry parameters, dated no more than ten days from the date of fecal examination, were included when requested by the clinician, either due to some health concern or routine testing as part of a preventive health management plan. It was not available in the database whether the fecal sample was submitted due to clinical signs of parasitism or not.

The date of testing was used to create season of testing, according to 3-month categories following the astronomical calendar: winter was from December 21st to March 20th; spring was from March 21st to June 20th; summer was from June 21st to September 20th; and autumn was from September 21st to December 20th. The variable “age” was divided into two categories: ≤ 1-yr-old and > 1-yr-old. Since the main part of the samples were from the Bologna province, the variable “geographic origin” was dichotomized as Bologna province or other provinces within Italy.

Overall, three datasets were used for the analysis. One comprehensive of the total number of dogs submitted to the VTH with a performed fecal parasitological exam in the study period, and two others, as subsets of the first one, including only dogs that had, respectively, a CBC or biochemical profile dated no more than ten days from the fecal examination. The potential effect of testing endoparasite-positive on specific CBC and biochemical parameters was investigated, such as serum total proteins, serum albumin, serum globulin, albumin-to-globulin ratio, C-reactive protein (CRP), and serum cobalamin.

The Shapiro-Wilk test was utilized to assess the distribution of continuous variables. Year, sex, age, geographic origin, and seasonality were examined, as putative risk factors for being diagnosed positive for at least one endoparasite and for the commonly isolated endoparasites, using Pearson’s χ^2^ test of independence. When low numbers of parasitized dogs (<5) of a particular genus/species, the Fisher’s exact test was utilized. Student’s t-test and Kruskal-Wallis non-parametric test were used to compare continuous variable results by parasitism status, for variables that were normally and non-normally distributed, respectively (e.g., blood parameters). Multivariable logistic regression analysis was also performed, using combined test results (endoparasite presence/absence) as the outcome variable. Backward stepwise regression was employed for the model building, and model fitness was assessed by means of the Hosmer-Lemeshow goodness-of-fit test. Testing for interaction and confounding of model variables was done [23]. Odds ratios (OR) and their 95% confidence intervals (CIs) were reported. Stata Statistical Software (Release 15, College Station, TX: StataCorp LLC) was utilized for the descriptive statistical analysis and the model building, analyses, and evaluations. Results were considered significant when P≤0.05.

## Results

A total of 1,972 fecal samples from privately owned dogs were processed to detect endoparasites. Based on the clinicians’ requests, a subset of the fecal samples, specifically 138 and 689, were additionally checked for lungworms by Baermann technique, and for *Giardia* trophozoites by direct smears with Lugol’s iodine staining and cysts by a double sedimentation, respectively. Sample submission information is summarized in Table 1.

**Table 1:** Descriptive statistics of predictors, by category, for 1,972 dogs tested for endoparasites at the Department of Veterinary Medical Sciences (Unibo) from 2011 to 2019.

Predictors	Categories	# of animals (%)
**Age**	≤ 1-yr	443 (22.5)
	> 1-yr	1,529 (77.5)
	Total	1,972
**Sex**	Male	890 (45.1)
	Female	526 (26.1)
	Castrated male	148 (7.5)
	Sterilized female	408 (20.7)
	Total	1,972
**Geographical origin**	Bologna (province)	1,452 (73.6)
	Other	520 (26.4)
	Total	1,972
**Season**	Winter	480 (24.3)
	Spring	589 (29.9)
	Summer	414 (21.0)
	Autumn	489 (24.8)
	Total	1,972

On average, the frequency of samples submitted was 219.1 per year (SD = 107.1), with a range from 63 to 339. There were slightly more males than females, with most males left intact, whereas nearly half of females were spayed. Slightly more samples were submitted in the spring than the other months.

Table 2 shows the frequency for each endoparasite genus found in the dog population. The most common endoparasites detected were *Cystoisospora* spp., *Toxocara canis*, *Giardia duodenalis*, and *Trichuris vulpis*, with 3%, 2.8%, 1.6%, and 1.2% frequency, respectively. The majority (89.7%) of the positive dogs had single infections, 8.8% had double infections, 1.0% had triple infections, and one dog harbored four different endoparasite genera (*T*. *canis*, *T*. *vulpis*, *Crenosoma vulpis*, and *Ancylostoma* spp.).

**Table 2:** Frequency of endoparasites detected in 1,972 dogs diagnosed at the Department of Veterinary Medical Sciences (Unibo), during 2011–2019.

	Parasites	# of positive samples	Frequency %	95% CIs
**Nematoda**	*Toxocara canis*	56	2.8	2.1–3.7
	*Trichuris vulpis*	23	1.2	0.7–1.7
	Ancylostomatidae	20	1.0	0.6–1.6
	*Eucoleus* sp.	13	0.7	0.3–1.1
	*Toxascaris leonina*	5	0.25	0.0–0.6
	*Angiostrongylus vasorum*	3	0.2	0.0–0.4
	*Strongyloides* sp.	2	0.1	0.0–0.4
	*Crenosoma vulpis*	1	0.05	0.0–0.3
	*Filaroides* sp.	1	0.05	0.0–0.3
**Protozoa**	*Cystoisospora* spp.	59	3.0	2.3–3.8
	*Giardia duodenalis*	32	1.6	1.1–2.3
**Cestoda**	Taenidae eggs	2	0.1	0.0–0.4
	*Dypilidium caninum*	2	0.1	0.0–0.4

Table 3 shows the frequency of animals tested positive by year. The proportion of animals found to be infected (eggs, cysts, oocysts, L1, and trophozoites) with at least one endoparasite genus was 10%. No significant difference was identified in overall positivity rates across the years (P = 0.704), and for lungworms (P = 0.160) or gastrointestinal parasites (P = 0.605) specifically.

**Table 3:** Frequency of tested and test-positive dogs for endoparasites, by year, diagnosed at the Department of Veterinary Medical Sciences (Unibo).

Year	# of tested animals	# of dogs tested positives	Frequency %	95% CIs
**2011**	63	5	7.9	2.6–17.5
**2012**	69	12	17.4	9.3–28.4
**2013**	154	15	9.7	5.5–15.5
**2014**	194	19	9.8	6.0–14.9
**2015**	280	29	10.3	7.0–14.5
**2016**	325	31	9.5	6.6–13.3
**2017**	322	28	8.7	5.9–12.3
**2018**	339	34	10.0	7.0–13.7
**2019**	226	25	11.1	7.3–15.9
**Total**	1,972	198	10.0	8.7–15.5

Table 4 shows the comparison between predictors by categories and dogs tested positive and negative for endoparasites. By comparing sex no difference in positive diagnoses was detected between male and female dogs, at 9.3% and 10.8%, respectively (P = 0.279). Sex was not a significant predictive factor for endoparasites even for the three most common endoparasite taxa detected, *Cystoisospora* spp. (P = 0.296), *T*. *canis* (P = 0.219), and *G*. *duodenalis* (P = 0.170).

**Table 4:** Pearson’s chi-square results for factors associated with endoparasite presence in 1,972 dogs diagnosed at the Department of Veterinary Medical Sciences (Unibo) from 2011 to 2019.

Predictors	Categories	# of dogs tested positive (%)	95% CIs	P-values
**Age**	≤ 1-yr	115 (26.0)	21.9–30.3	<0.001
	> 1-yr	83 (5.4)	4.3–6.7	
**Sex**	Male	97 (9.3)	7.6–11.3	0.279*
	Female	101 (10.8)	8.9–13.0	
**Geographical origin**	Bologna (province)	158 (10.9)	9.3–12.6	<0.001
	Other	40 (7.7)	5.6–10.3	
**Season**	Winter	71 (14.8)	11.7–18.2	<0.001
	Spring	47 (8.0)	5.9–10.4	
	Summer	23 (5.6)	3.6–8.2	
	Autumn	57 (11.7)	8.9–14.8	

* No statistical significance; male included bother intact and castrated, while female included both intact and sterilized, in an effort to reduce degrees of freedom to enhance statistical significance.

As for age classes, 26% of ≤ 1-yr-old dogs was diagnosed positive for fecal endoparasites while dogs older than 1-yr-old showed a proportion of positive diagnoses of only 5.4% (P<0.001). The chance of testing positive in our dog population was 6 times higher in ≤ 1-yr-old dogs than >1-yr-old ones (OR = 0.164; P<0.001). By measuring the effect of the age, expressed as a continuous variable (by month) on the three main genera, for each increased month of age, the probability to have a dog positive to *Cystoisospora* spp. decreased by 5% (OR = 0.95; P<0.001), 1.7% for *T*. *canis* (OR = 0.984; P<0.001), and finally 6% less probability for each month increased for *G*. *duodenalis* (OR = 0.94; P<0.001).

The proportion of dogs diagnosed positive for at least one endoparasite genus, by geographic origin, differed significantly (P<0.001), as dogs from Bologna province showed a frequency of 10.9% compared to other dogs with 7.7%.

Looking to the effect of the seasons of testing positive for endoparasites, 14.8% of coprological exams performed in winter resulted in positive tests, 11.7% in autumn, 8.0% in spring, and 5.6% during summer (P<0.001). The chance of being test-positive during winter was nearly 2 times higher than in spring (OR = 0.502; P = 0.001), and 3 times higher compared to summer (OR = 0.340; P<0.001), but not statistically different than autumn. As for the main endoparasite genera detected, determination of Pearson’s χ^2^ test showed a seasonal pattern only for *G*. *duodenalis*, most frequently diagnosed in winter compared with other seasons (P = 0.001). *G*. *duodenalis* presented 7 times higher probability to be diagnosed in winter compared to summer (OR = 0.141; P = 0.009) and 5 times higher compared to spring (OR = 0.198; P = 0.004). Conversely, no difference was found in probability to have positive dogs between winter and autumn (P = 0.219).

Based on our 1,972 dog observations with data for the final multivariable logistic regression model (controlling for confounding—Table 5), dogs less than or equal to 1-yr-old was a risk factor for the diagnosis of parasitic infections by a factor of 6.1 times compared to dogs older than 1-yr-old. Cold seasons increased the odds of being diagnosed endoparasite-positive by a factor of 2.03 compared to the warm seasons.

**Table 5:** Predictors of the final multivariable logistic regression model of factors associated with endoparasite presence in 1,972 dogs diagnosed at the Department of Veterinary Medical Sciences (Unibo) from 2011–2019.

Predictors	Odds Ratio	95%CI	P-value
**Age**			
> 1-yr	baseline	baseline	-
≤ 1-yr	6.106	4.482–8.317	<0.001
**Season**			
Warm season	baseline	baseline	-
Cold season	2.027	1.474–2.786	<0.001

* Variable season dichotomized: Warm season = spring+summer; Cold season = autumn+winter, in an effort to reduce degrees of freedom to enhance statistical significance

The second dataset included 1,104 dogs with a fecal examination and a CBC completed no more than 10 days prior to the fecal examination. On average, the number of dogs that met these inclusion criteria were 122.7 per year (SD = 60.2), with a range from 36 to 197. Slightly fewer dogs had both a fecal examination and serum biochemistry conducted (n = 987 –the third dataset). Descriptive statistics and reference values for these blood and serum parameters for all dogs with blood results are shown in Table 6.

**Table 6:** Descriptive statistics of investigated clinicopathological parameters most frequently affected by endoparasite presence within the literature, regardless of endoparasite status.

Parameters	Minimum	Maximum	Mean or (Median)	SD or (IQR)	Reference Values
2^nd^ dataset with endoparasite data and CBC parameters in 1,104 dogs					
**Hematocrit (%)**	13.9	68.5	45.0	8.1	37–55
**Hemoglobin (g/dL)**	4.2	24.5	15.2	2.9	12–18
**RBCs (x10** ^**6**^ **/μL)**	2.2	10.4	6.6	1.1	5.5–8.5
**MCV (fL)**	47.3	103.4	68.2	4.0	60–77
**MCHC (g/dL)**	25.2	57.0	33.8	1.7	32–38
**Eosinophils (/μL)** ^**a**^	2	6601	(357)	(149–627)	0–750
**Leucocytes (/μL)**	250	59060	12415.3	6312.6	6000–17000
**Neutrophils (/μL)** ^**a**^	19	52800	(7119)	(5328–10218)	3000–12000
**Basophils (/μL)** ^**a**^	3	1068	(53)	(33–86)	0–180
**Lymphocytes (/μL)**	45	11316	2336.1	1187.3	1000–4800
**Monocytes (/μL)**	43	5768	766.2	616.9	100–1400
3^rd^ dataset with endoparasite data and serum parameters in 987 dogs					
**Total Proteins (g/dL)**	2.31	10.28	6.14	1.00	5.6–7.3
**Albumin (g/dL)**	0.95	4.50	2.95	0.58	2.75–3.85
**Globulin (g/dL)**	1.13	7.86	3.19	0.74	2.53–3.73
**A/G Ratio**	0.26	2.12	0.95	0.23	0.75–1.35
**CRP (mg/dL)**	0.01	35.81	3.61	5.85	0–0.85
**Cobalamin (ng/L)**	150	1000	473.23	275.23	250–730

^a^ Non-parametric variable.

CBC, complete blood count; RBCs, red blood cells; MCV, mean corpuscular volume; MCHC, mean corpuscular hemoglobin concentration; A/G, albumin/globulin ratio; CRP, C-reactive protein.

Table 7 summarizes CBC values obtained, by parasitological status. Values, on average, were significantly lower in parasitized dogs compared to those tested negative for MCHC, and significantly higher for basophils, lymphocytes, and monocytes. No differences were observed among positive and negative groups regarding total leucocytes, eosinophils, neutrophils and mean corpuscular volume (MCV) values. Additionally, Table 7 summarizes serum values obtained, by parasitological status. The albumin-to-globulin ratio, CRP, and cobalamin B12 levels were not different between positive and negative tested dog populations.

**Table 7:** Complete Blood Count parameters in the 1,104 eligible dogs and serum biochemical parameters in 987 eligible dogs, by positive and negative endoparasite groups, and relative P-values of Student’s t-tests (unless otherwise indicated).

Parameters (CBC)	Mean (Median) value in parasite positive group	Mean (Median) value in parasite negative group	P-values
**MCV (fL)**	67.8	68.3	0.227*
**MCHC (g/dL)**	33.3	33.8	0.005
**Eosinophils (/μL)**	(298)	(364)	0.117*^a^
**Neutrophils (/μL)**	(8591)	(8668)	0.890*^a^
**Basophils (/μL)**	(64)	(53)	0.025^a^
**Lymphocytes (/μL)**	2729.7	2295.6	0.004
**Monocytes (/μL)**	885.4	753.0	0.040
**Leucocytes (/μL)**	13145.24	12340.22	0.218*
**Parameters (biochemistry)**	**Mean value in parasite- positive group**	**Mean value in parasite-negative group**	
**A/G Ratio**	0.938	0.957	0.472*
**CRP (mg/Dl)**	3.7	3.6	0.665*
**Cobalamin (ng/L)**	460.2	474.5	0.858*

*No statistical significance

^a^ Kruskal-Wallis’ test P-value

MCV, mean corpuscular volume; MCHC, mean corpuscular hemoglobin concentration; A/G, albumin/globulin ratio; CRP, C-reactive pro.

Table 8 summarizes CBC and serum values of the age-dependent parameters analyzed in dogs aged ≤ 1-yr and > 1-yr. We used 12 months as the cutoff because it made more biological sense (i.e., when dichotomizing at 6 months, the parasitized dogs over 6 months had lower parameters which does not make sense) and 12 months was used elsewhere [3]. Hematocrit, hemoglobin, RBCs, total protein, and albumin values revealed a significant difference in mean values in endoparasite positive (lower) versus negative (higher) dogs ≤ 1 year old.

**Table 8:** Age-dependent parameters by positive and negative endoparasite groups stratified by age [3], and relative P-values of Student’s t-tests.

**Parameters (CBC)**	**Age**	**Mean value in parasite positive group**	**Mean value in parasite negative group**	**P-values**
**Hematocrit (%)**	≤ 1-yr	37.1	41.2	<0.001
	> 1-yr	44.7	46.0	0.23*
**Hemoglobin (g/dL)**	≤ 1-yr	12.2	13.7	<0.001
	> 1-yr	15.1	15.6	0.161*
**RBCs (x10** ^**6**^ **/μL)**	≤ 1-yr	5.59	6.17	<0.001
	> 1-yr	6.52	6.73	0.199*
**Parameters (biochemistry)**	**Age**	**Mean value in parasite positive group**	**Mean value in parasite negative group**	
**Total Protein (g/Dl)**	≤ 1-yr	5.09	5.64	0.001
	> 1-yr	6.09	6.28	0.198*
**Albumin (g/Dl)**	≤ 1-yr	2.48	2.87	<0.001
	> 1-yr	2.88	2.99	0.208*

* No statistical significance.

CBC dataset: 50 (27.5%) out of 182 dogs aged ≤1-yr tested positive and 53 (5.75%) out of 922 > 1-yr.

Serum dataset: 39 (24.5%) out of 159 dogs aged ≤1-yr tested positive and 48 (5.8%) out 828 > 1-yr tested positive.

All parameters are reported as the mean.

RBCs, Red Blood Cells.

## Discussion

This long-term large retrospective study describes the frequency of endoparasites, diagnosed through fecal exam, in owned dogs submitted to the VTH (DIMEVET-Unibo) during a nine-year period of diagnostic activity, exploring potential risk factors and seasonal patterns, and combining fecal parasite results with hematological parameters whose alterations are sometimes attributed to the presence of endoparasites.

The number of visits to veterinary clinics per year shows a gradually positive trend, which may be due to both the increasing number of pet dogs in Italy in the last decade and greater perception of the benefit of regular pet care [1].

Although our study population does reflect a well-cared proportion of owned dogs, endoparasites are still present, with 10% of positive dogs infected with at least one parasite species. The frequency of infection is 3-fold lower than reported by Scaramozzino et al. [24], in a study which also included shelter dogs. This difference in frequency likely reflects the lack of regular veterinary care and deworming programs afforded to shelter dogs [25]. This care gap is even more prominent in stray dogs where a parasite prevalence of 69% was found in examined stray dogs in Central Italy [26].

In the current study, two of the most frequently detected endoparasites are zoonotic or potentially zoonotic, *T*. *canis* and *G*. *duodenalis*, respectively. This is consistent with other studies conducted on well-cared-for, owned dogs [3, 27–29]. *T*. *canis* is estimated to infect tens of millions of people annually [30]. This frequent infection is not surprising given that over 100 million dogs worldwide are estimated to be infected with *T*. *canis*, and that they shed billions of eggs into the environment annually [31]. Also, *G*. *duodenalis* infects a wide range of hosts, including humans, domestic, and wild mammals [32]. The potential dog-to-human transmission of *Giardia* has been well studied. To date, only two (A and B) of the eight major genetic assemblages have been isolated from humans and animals; with humans being more commonly infected with the Assemblage A subtype II, whereas dogs are predominantly infected with subtype AI [33]. Subtype AII has been found in dog samples from several countries [34–38]. For this reason, when considering the zoonotic potential of *Giardia* assemblages, we should remain cautious until studies consistently incorporate more sensitive subtyping tools [39]. The epidemiology and the risk associated with *Giardia* infection for humans are challenging to evaluate, as sophisticated tools and high human-expertise are required. Furthermore, most studies report results originating from DNA detection in fecal samples only, without complementary microscopic evidence of cysts or trophozoites. Therefore, without supporting evidence, it is difficult to confirm that these hosts were infected and not simply passaging cysts, or even ingested DNA, within their feces [40].

The helminth parasites of the respiratory tract are sporadically or even rarely detected roundworms in dogs [41]. The most common species detected include *Eucoleus* spp., *Angiostrongylus vasorum* (the French heartworm), and *Crenosoma vulpis* (the Fox lungworm). No significant increase in the number of dogs detected positive for these parasites occurred through the study period, but in the last two years of observation, the frequency was over 1–2%. These parasites may have a life-threatening potential effect in dogs sharing a high degree of environmental overlap with foxes [42], the main reservoir, but also in dogs living in urban and suburban areas as they are considered re-emerging parasites in pets. Additionally, unsuccessful attempts to treat dogs with different anthelmintic drugs for *Eucoleus* spp. are sporadically reported [43, 44], posing a real threat of infection in dogs.

The current study indicates that the annual endoparasite frequency has remained constant. Few studies take into account changing yearly patterns in parasite prevalence. Barutzki and Shaper [45], speculate over minor variation through the 7-year study period in Germany without statistical differences, even if their overall proportion of positive animals were higher than the current study. On the contrary, an 18-year retrospective study in Canada demonstrated a decreasing odds of positivity of around 4% for each additional year within the database [3]. Finally, “a subtle fluctuation in prevalence for roundworms over a 7-year period” was noted in a US study [46], describing an increasing prevalence for *Ancylostoma* spp., and a slightly decreasing prevalence for *Trichuris vulpis*.

Age of susceptibility is an important predictor for endoparasites [47]. In the current study, the chance of being positive for at least one parasite was 6 times higher in dogs less than or equal to 1-yr-old versus dogs over 1-yr-old. Numerous studies have documented a similar likelihood for this age group [3, 28, 48]. The wide difference in decreasing percentage probabilities month-by-month for *T*. *canis* compared to *G*. *duodenalis* and *Cystoisospora* spp. maybe due to the longer patent period for the roundworm compared to protozoa. Indeed, patency of > 90 days has been reported by Fahrion et al. [49]; a patent period of this length may imply an average life span of *T*. *canis* worms of 4 months [50].

Gender is an often-explored predictor in epidemiological studies on endoparasite distribution; however, its predictive meaning on the parasites’ presence in dogs is likely null. In the current study, no statistical significance was calculated when sex was considered as a dichotomous variable (male, female) (P = 0.279), instead the sexually intact dogs were significantly more parasitized compared to neutered dogs (P<0.001). This association suggests that age can be a confounding factor on sexual status since the median ages of intact patients was significantly lower compared to neutered patients in the study.

Differences in the endoparasite-positive proportion among dogs from Bologna province (10.9%) compared to dogs from outside the region (7.7%) is probably because the DIMEVET-VTH acts as a referral clinic. Dogs are typically referred as patients for specialist services and coprological exams are not always requested or are directly performed by private veterinary clinics.

In the current study, a seasonal pattern in endoparasite frequency was noted, as submitted samples significantly harbored parasites more frequently during winter and autumn (P<0.001) than the other two seasons. Specifically, the odds that dogs harbored fecal parasites were 2- and 3-time higher in winter than in spring and summer, respectively. This seasonal association may reflect an increased likelihood of exposure during summer when dogs are free to spend more time outside [3], or it could be related to evolutionary canid reproduction cycles and parasite/host co-evolution [46]. Many helminths are transmitted transovarially or transmammary (e.g *Toxocara* and *Ancylostoma* respectively) [51]. Therefore, with two heat cycles per year and an increase in frequency of pups being born in the early spring and late autumn, more pups are likely to be detected with parasites in winter.

Among the three most commonly detected endoparasites, only *G*. *duodenalis* showed a significant seasonality (P<0.001), being diagnosed 7- and 5-time more frequently in winter and spring as compared to summer. This seasonality pattern for *G*. *duodenalis* has previously been reported in Italy by Bianciardi et al. [10]. A reasonable explanation for the increased diagnoses of *G*. *duodenalis* during winter is that environmental conditions (relative humidity and lower temperature) favor the parasites survival and transmission compared to the drier summer season at our latitude [52, 53].

The multivariable logistic regression shows that when the variable “age” is added to the model with “geographical origin” and “sex”, these variables become non-significant showing that they are not really risk factors because they are confounded by “age”. Thus, our study shows that age is the main predictor for the diagnoses of intestinal parasites in dogs, and the cold seasons also increase the chance for dogs of being diagnosed parasite-positive in Italy, even among dogs of similar age.

Several studies have reported variation in blood count parameters, primarily associated with specific endoparasites, such as *Ancylostoma* spp. and *Angiostrongylus vasorum* [54, 55]. In the present study, although the positive dog population seems to affect mainly red blood cell parameters (hematocrit, hemoglobin, MCHC and RBCs), the values were within reference interval values, which is opposite to other studies [17, 56, 57]. However, these nonspecific signs are not always manifested, even when considering *Ancylostoma* spp. a blood-sucking nematode [55]. Despite the pivotal role of eosinophils [58], basophils [59, 60] and neutrophils [17] in the immune response to parasite infection, our results showed no significant change in either parameter. This difference in results could also be explained by low parasite burdens that were unable to generate a strong immune response. Unfortunately, information on the clinical status, history of previous exposure and quantitative parasitological results were unavailable in the dataset.

The overall serum protein profile in the endoparasite-positive dog group appears slightly lower than the parasite-negative dog group. Under conditions of moderate parasite infection, plasma proteins may undergo little change. The clinical signs, laboratory findings, and severity of the disease are likely related to the intensity of the parasite infection (i.e., the greater the parasite load in the small intestine, the more severe the hypoalbuminemia produced). The endoparasite burden within the infected dog population may not have been sufficiently high to justify an evident increased CRP nor decreased cobalamin level. Further research is needed to quantitatively assess how the burden of infection may affect these serum parameters. Given the subtle differences between infected and uninfected dogs and the low degree of specificity of these changes for endoparasitic infection, clinicopathologic parameters cannot replace regular fecal screening.

The current results were retrospectively analyzed and since they originated from diagnostic laboratories could contain selection biases, and limitations, as they reflect the situation for a well-cared dog population that was not randomly selected. However, the study period (9-years) offers an overview about the temporal frequency of which endoparasites are diagnosed in dogs. Despite the selection biases, the results of the risk factor analyses may still reflect relationships found in a randomly selected population, as well as the effect of parasites on blood parameters. Furthermore, the samples submitted to the VTH included both clinically normal animals and those exhibiting clinical signs of disease, as clinicians sometimes (based on their clinical judgement, not consistently) requested diagnostics for *Giardia* and lungworms based on clinical signs of diarrhea or coughing, respectively; therefore, the frequency related to these parasites could have also been under-estimated.

Another limitation of the study is the imperfect sensitivities of the tests utilized by the laboratory, especially the testing for *Giardia* where an optical microscope and Lugol’s iodine staining solution were used, which could lead to under-estimates of the frequency of infection. Despite this information bias, we strongly encourage veterinarians to collect and mix multiple stool samples per dog before diagnostics are performed in order to increase sensitivity. Unfortunately, we were not able to recover such information for fecal flotation, but for the Baermann technique, the laboratory requires the submission of three-day consecutive samples prior to conducting the test. Future research should consider the impact of parasite burden on host clinicopathological outcomes. Finally, having datasets with information regarding the health status of dogs would have been useful in interpreting the blood parameters but that was not possible for our study.

## Conclusion

This study provides an impression of the parasitic infection status in fecal samples from well-cared dogs brought to a veterinary college during a nine-year period. Although retrospective long-term longitudinal studies may lack statistical robustness, they provide insight on the changes in infectious disease over time, thereby offering private veterinarians and public health officials relevant knowledge to manage and mitigate transmissible diseases. The study found a constant and fairly high frequency of endoparasite infection in well-cared-for dogs. Two of the three most common endoparasites diagnosed are zoonotic or potentially zoonotic, and this illustrates the crucial role of veterinarians in educating owners about the associated zoonotic risk. From the study, parasitic infection is dependent on age and season, and knowing these risk factors may help clinicians to program specific anthelmintic protocols to reduce the risk of transmission. Overall, blood and serum protein parameters only showed moderate evidence of endoparasite infection.
